# Bloch Equations-Based Reconstruction of Myocardium T1 Maps from Modified Look-Locker Inversion Recovery Sequence

**DOI:** 10.1371/journal.pone.0126766

**Published:** 2015-05-11

**Authors:** Benjamin Marty, Alexandre Vignaud, Andreas Greiser, Benjamin Robert, Paulo Loureiro de Sousa, Pierre G. Carlier

**Affiliations:** 1 Institute of Myology, NMR Laboratory, Paris, France; 2 CEA, DSV, I2BM, MIRCen, IdM NMR Laboratory, Paris, France; 3 UPMC University Paris 06, Paris, France; 4 CEA, DSV, I2BM, NeuroSpin, UNIRS, Gif-sur-Yvette, France; 5 Siemens AG Healthcare Sector, Erlangen, Germany; 6 Siemens Healthcare, Saint Denis, France; 7 Université de Strasbourg, CNRS, ICube, FMTS, Strasbourg, France; University Hospital of Würzburg, GERMANY

## Abstract

Modified Look-Locker Inversion recovery (MOLLI) sequence is increasingly performed for myocardial T1 mapping but is known to underestimate T1 values. The aim of the study was to quantitatively analyze several sources of errors when T1 maps are derived using standard post-processing of the sequence and to propose a reconstruction approach that takes into account inversion efficacy (η), T2 relaxation during balanced steady-state free-precession readouts and B1+ inhomogeneities. Contributions of the different sources of error were analyzed using Bloch equations simulations of MOLLI sequence. Bloch simulations were then combined with the acquisition of fast B1+ and T2 maps to derive more accurate T1 maps. This novel approach was evaluated on phantoms and on five healthy volunteers. Simulations show that T2 variations, B1+ heterogeneities and inversion efficiency represent major confounders for T1 mapping when MOLLI is processed with standard 3-parameters fitting. *In vitro* data indicate that T1 values are accurately derived with the simulation approach and *in vivo* data suggest that myocardium T1 are 15% underestimated when processed with the standard 3-parameters fitting. At the cost of additional acquisitions, this method might be suitable in clinical research protocols for precise tissue characterization as it decorrelates T1 and T2 effects on parametric maps provided by MOLLI sequence and avoids inaccuracies when B1+ is not homogenous throughout the myocardium.

## Introduction

Cardiac magnetic resonance (CMR) imaging is currently one of the most popular modalities to characterize myocardial tissue. Indeed, T1-, T2- and T2*-weighted images, alone or T1 weighting combined with contrast agent injection, are capable of revealing multiple structural abnormalities such as edema [1], fibrosis [2,3], as well as iron overload [4]. Lately, the increase in signal-to-noise ratio with high-field MRI scanners as well as the development of powerful gradient systems and parallel imaging reconstruction has opened the way to quantitative CMR (see [5] for review). In particular, measurements of myocardium T1 and T2 relaxation times are extending the range of tissue parameters that could be assessed to monitor natural history of disease or therapy response. It has also been demonstrated that quantification of myocardial T1 relaxation times enables detection of diffuse lesions [2,6], or even quantification of extracellular volume fraction [7].

Over the last decade, a new method called MOLLI (Modified Look-Locker Inversion recovery) has been developed and is increasingly performed for myocardial T1 mapping [8]. The sequence consists of the acquisition of several balanced steady-state free-precession (b-SSFP) images at different times (TI) following magnetization inversion. The b-SSFP images are acquired in a single breath-hold, over several cardiac cycles, for the same cardiac phase at end of diastole. This sequence was successfully used to reveal diffuse increased volume of distribution of gadolinium-chelate in patients suffering from cardiomyopathies [9] and amyloidosis [10]. Furthermore, MOLLI sequence enabled detection of subtle T1 relaxation time changes without contrast agent injection in pathologies such as acute myocardial infarction [11,12] or Anderson Fabry disease [13].

Although the MOLLI method has been widely used for quantitative T1 mapping, it is also known to underestimate high T1 values (more than 15% underestimation for T1 values higher than 1500ms) [14]. In a recent publication by Gai et al. [15], the MOLLI sequence was characterized using a step-by-step numerical solution of Bloch equations. Authors showed that the variation of parameters such as T2, nominal flip angle or k-space sampling trajectory could have a significant impact on the accuracy of T1 quantification. Discrepancies were evaluated in a phantom exhibiting different T1 and T2 values. Several contributions to the T1 underestimation were found, e.g. the effect of T2 during b-SSFP acquisition is not considered in the standard post-processing of the sequence. Other key factors, such as the encoding scheme for b-SSFP acquisitions, inversion efficiency and B1+ inhomogeneities through the myocardium can also impact the accuracy of T1 measurement. Thus, the T1 map estimation out of MOLLI data would be largely improved by taking into account T2, B1+ effects and inversion imperfection. In particular, estimation of parameters like extracellular volume fraction would greatly benefit from a more accurate quantification method.

The aim of the present study was first to use Bloch equations simulation to precisely quantify the errors made when T1 maps are derived using standard post-processing of the MOLLI sequence. Then, we proposed a robust approach to derive more accurate T1 maps using MOLLI data, fast T2 and B1+ maps (compatible with CMR) and Bloch equations simulation. This novel approach was evaluated on phantoms and compared to the standard post-processing method on five healthy volunteers.

## Methods

### MOLLI sequence and standard post-processing approach

The acquisition scheme of the MOLLI sequence consists of three blocks of 3, 3, and 5 single-shot b-SSFP images that are acquired at different time points after magnetization inversion. The sequence is ECG triggered, and the acquisition of each image is performed during the same phase of cardiac period, at the end of diastole. The acquisition of each block is followed by a resting period of three R-R cycles allowing for longitudinal magnetization relaxation.

In the standard post-processing approach of this sequence, T1-fitting of the signal (S) is performed by a non-linear least-square adjustment to an exponential recovery curve:

S(TI)=A−Be−TIT1* (1)

where TI is the inversion time of each acquired image. T1* is the effective relaxation constant which is modified from T1 because of image readout. T1 is then calculated using the following equation [8]:

$$T_{1}=T_{1}^{*}\left(\frac{B}{A}-1\right)$$ (2)

This Look-Locker correction can be derived analytically in the case of a FLASH readout with continuous RF [16]. However, unlike typical Look-Locker scans, MOLLI is acquired using non-continuous b-SSFP readouts, resulting in intermittent free recovery of magnetization. As the longitudinal recovery curve has modulated and unmodulated parts, MOLLI exhibits a complex response to scan and intrinsic tissue parameters (nominal flip angle, inversion efficiency, T2) that are not properly modeled in this standard approach.

### Simulation-based post-processing of MOLLI

Bloch equations simulation of the MOLLI sequence was implemented using Matlab (The Mathworks, USA) as described in [17] and [15]. Magnetization was described step by step using a general matrix representation:

$$M_{k+1}=AM_{k}+B$$ (3)

where **M**_**k**_ is a 3D vector representing magnetization at the k^th^ step of the sequence, **A** and **B** are respectively transformation matrix and vector that take into account RF pulses, free precession, longitudinal and transverse relaxation. The equilibrium magnetization is **M**_**0**_ = [0 0 1]^T^.

Due to magnetization decay during the application of the adiabatic inversion pulse, the inversion efficiency (η = cos(180—inversion pulse)) is commonly reduced [18].

Using acquisition parameters typically used for MOLLI, the b-SSFP readout consists of 5 preparation pulses (α/6,-α/3, α/2, -2α/3, 5α/6) separated by TR, followed by a 69 ±α pulses train to read the k-space in a linear manner. During this readout period, Eq 3 becomes:

$$M_{k+1}=P_{1}C_{1}R_{\alpha }P_{2}C_{2}M_{k}+P_{1}C_{1}R_{\alpha }D_{1}+D_{2}$$ (4)

where **P**_**1,2**_ **, C1,2, D1,2, and Rα** are taking into account RF pulses, free precession and T1 and T2 relaxation (see further details in Appendix). For the first block of three images, TI_1_ corresponds to the duration between the inversion and the acquisition of the central line of the k-space of the first image. The two other images of the block are sampled after one and two R-R intervals, respectively. For the second and third blocks, TI_2_ and TI_3_ are defined as TI_2_ = TI_1_ + ΔTI and TI_3_ = TI_1_ + 2×ΔTI.

During resting periods, only free precession and relaxation were taken into account for magnetization evolution. For given T1, T2, inversion efficacy η and flip angle α, the simulation returns a time dependent function f(T1, T2, η, α,t), that is determined at the 11 inversion times of the MOLLI sequence.

This simulation was used to emphasize the deviation between true T1 and T1 values estimated by the standard post-processing approach as a function of T1, T2, nominal flip angle (α), and inversion efficacy η. Four different datasets were simulated using actual timings of the MOLLI sequence, and varying either T1, T2, α or η (Table 1). For each set of parameters, the 11 MOLLI points were reordered as a function of TI and the 3-parameter model corresponding to Eq 1 was adjusted to the recovery curve. T1 was then derived from Eq 2 and compared with the true T1.

**Table 1:** T1, T2, α and η parameters used to generate the four different datasets using Bloch equations simulation.

**Dataset**	**T1 (ms)**	**T2 (ms)**	**α (°)**	**η**
**1**	Varying: 400–2400	60	35	1
**2**	1180	Varying: 20–100	35	1
**3**	1180	60	Varying: 5–75	1
**4**	1180	60	35	Varying: 0.85–1

**Fig 1:**
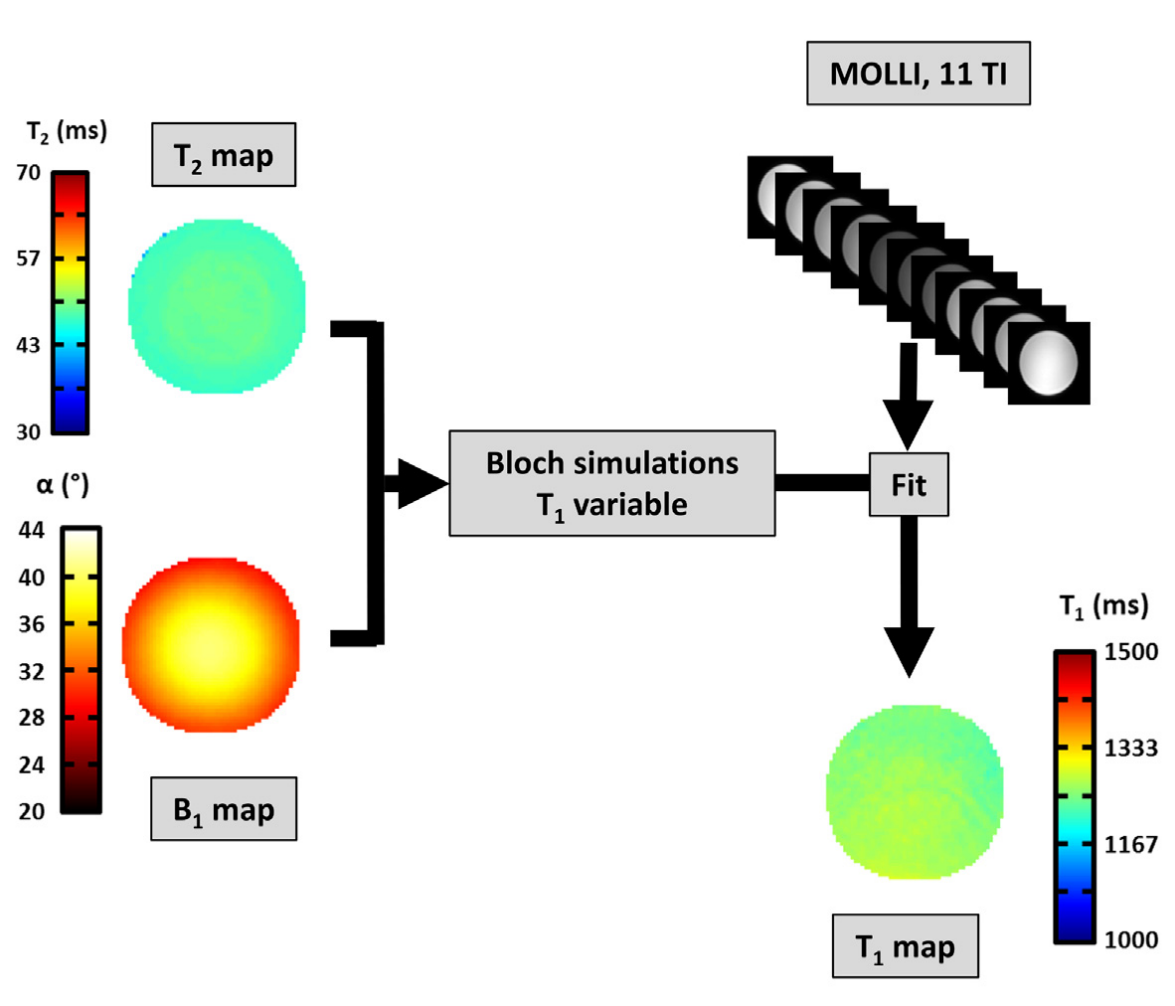
Description of the novel post-processing approach of the MOLLI sequence to derive T1 maps on a 2.2L agar phantom. Simulated datasets were adjusted pixel by pixel on experimental data points obtained using the MOLLI sequence by varying T1 and using T2 and B1+ parameters measured with separate specific MR sequences.

A novel post-processing procedure was then proposed to derive a more accurate T1 map from experimental MOLLI data. First, inversion efficiency was evaluated using Bloch simulation of the hyperbolic secant pulse as well as B0 and B1+ maps. A T2 map was then generated using an additional specific sequence. Finally, for each pixel of the MOLLI acquisition, the function f was fitted on the 11 experimental data points with T1 considered as a free parameter and T2, η and α as fixed parameters (Fig 1). The adjustment was performed by an unconstrained nonlinear optimization algorithm based on the Nelder-Mead Simplex Method [19].

### Monte Carlo simulation

A Monte Carlo simulation was implemented to quantify the influence of T2 and B1+ measurements on T1 quantification. A large set of data (10^5^) was generated using the Bloch equations simulation and standard *in vivo* parameters: nominal flip angle α_nom_ = 24°, T2_nom_ = 42 ms, 400 < T1 < 2400 ms and a simulated 60 b.p.m. heart rate. For each dataset, the 11 points of the MOLLI sequence were generated, including a noise corresponding to the experimental SNR. Datasets were then processed either by the standard or the novel post-processing approach using modified T2 and flip angle values (T2_mod_ and α_mod_). These modified values were generated from nominal values and mean standard deviations of T2 and B1+ mapping sequences (std_T2_ = 3ms and std_α_ = 2.5° respectively) observed in vivo through the septum:

$$T2_{mod}=T2_{nom}+randn\left(std_{T2}\right)$$ (5)

$$\alpha_{mod}=\alpha_{nom}+randn\left(std_{\alpha }\right)$$ (6)

where randn(std_T2_) and randn(std_α_) are random values picked up in normal distribution of standard deviations std_T2_ and std_α_ respectively.

### Phantoms

Inversion efficacy was determined on a 2.2L agar phantom (0.3%, w/w) doped with 0.64g/L NiCl2 mimicking relaxation times of the myocardium. The novel T1 mapping reconstruction method was evaluated on a phantom containing six agar tubes (0.3% w/w) doped with different concentrations of Gd-DOTA (Guerbet, France): (0.01, 0.03, 0.05, 0.1, 0.2, and 0.3 mM_Gd_). Longitudinal relaxation times of the six tubes were also measured using a gold standard inversion recovery turbo spin echo (IR-TSE) sequence. *In vivo* T2 mapping sequence was validated on a phantom containing five agar tubes (0.1, 0.2, 0.3, 0.4 and 0.5% w/w) doped with 0.05mM of Gd-DOTA (Guerbet, France). A cardiac rate of 60 beats per minute was simulated for MR imaging.

### Volunteers

The study was approved by the local ethics committee (Comité de Protection des Personnes (CPP) Ile de France VI) and written informed consent was obtained from all subjects. Five healthy men (mean age: 27.4 ± 8.6 years) with no history of cardiovascular or systemic disease were enrolled in the protocol. Image acquisition was ECG-triggered and performed during breath-holds on standard apical, medial and basal short-axis sections of the heart.

### MRI experiments

MRI experiments were carried out on a 3 T Trio Tim system (Siemens AG Healthcare Sector, Erlangen, Germany) with a 60 cm inner diameter and equipped with a 45 mT/m whole-body gradient system. RF transmission was performed by a body coil, and a 32-channel receiving heart coil (Rapid Biomedical, Wurzburg, Germany) was used for both phantom and *in vivo* imaging.

For *in vitro* imaging, baseline T1 values were assessed using a standard IR-TSE sequence: TE_eff_/TR = 15/11000 ms, 24 inversion times (from 30 to 8000 ms), matrix = 128 × 102, turbo factor = 7. Baseline T2 values were estimated using a spin echo sequence repeated at different echo times with the following parameters: TR = 3000 ms, 8 TE (10, 20, 40, 60, 90, 120, 200, 400 ms), matrix = 192 × 154. The prototype MOLLI sequence was acquired with a standard implementation consisting of 3 inversion sets of 3, 3 and 5 images, TE/TR = 1.25/2.5 ms, nominal flip angle = 35°, TI_1_ = 100 ms, ΔTI = 80 ms, matrix = 192 × 154, FOV = 410x330 mm^2^, BW = 180000 Hz, slice thickness = 6 mm, GRAPPA = 2 with 24 integrated reference lines, 75% of partial Fourier, 3 R-R cycles recovery period and acquisition time = 17 R-R cycles. MOLLI was also acquired with the same parameters but recovery period before inversion varying between 0 and 8 R-R cycles, leading to acquisition time of 11 to 27 R-R cycles. For *in vivo* T2 mapping, because of the limited acquisition window available during breath-hold, the standard SE sequence could not be performed and was replaced by a T2-prepared b-SSFP [20]. Sequence parameters were the following: TE/TR = 1.3/2.6 ms, 3 T2-preparation times (0/25/75 ms), 3 R-R cycles recovery periods, matrix = 192 × 154, FOV = 410x330 mm^2^, BW = 180000 Hz, slice thickness = 6 mm, acquisition time = 12 R-R cycles.

B1+ transmit field maps were obtained using Bloch-Siegert method [21]. An off-resonance Fermi pulse (Δω = ±8 kHz, duration = 8 ms, flip angle = 600°) was added inside a standard gradient-recalled echo (GRE) sequence (TE/TR = 12/25 ms, matrix = 128 x 64) to make phase signal sensitive to the transmit field. The two acquisitions (± Δω) necessary for B_1_+ map reconstruction were interleaved to reduce motion artifacts and the sequence was segmented (4 segments per shot) to reduce echo train duration to 200 ms. The total acquisition time of the Bloch-Siegert sequence was 16 R-R cycles.

B0 maps were also acquired on phantoms and on one of the five volunteers with a segmented dual gradient echo sequence, TE1/TE2/TR = 1.9/4.7/6.9ms, matrix = 128x128, 75% of partial Fourier, slice thickness = 6 mm, acquisition time = 3 R-R cycles. All the sequence acquired *in vivo* were performed under breath-hold. T2-prep and Bloch-Siegert images were registered on the 11 images of MOLLI sequence using an automatic rigid motion correction algorithm (MedINRIA, Version 1.9.4, France) based on the block matching method [22].

### Statistics

*In vivo* T1 values were analyzed in manually drawn regions of interest including the myocardial septum at the three imaging levels (basal, medial and apical). Student’s t-test was performed on the measured T1 to compare the two post-processing approaches. MOLLI sequence was also acquired *in vivo* without recovery period before second and third inversions. For each post-processing method, paired t-tests were employed to compare T1 values determined by both sequence settings. Differences were considered significant for P values <0.01.

## Results

### Simulations

**Fig 2:**
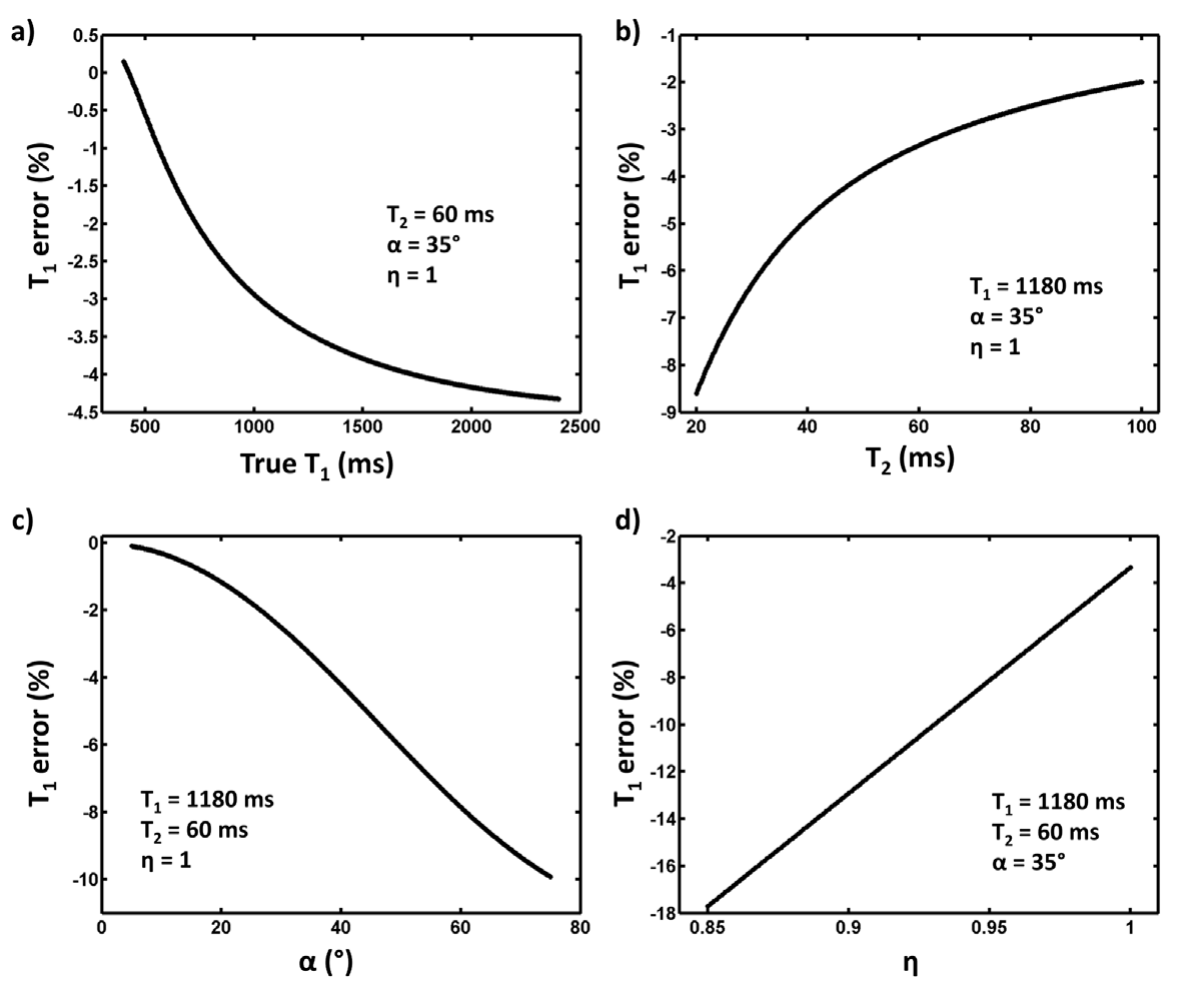
Deviation between true T1 values and T1 values determined by the standard post-processing approach as a function of T1 (a), T2 (b), nominal flip angle (c), and inversion efficacy η (d). The first dataset was generated using T2 = 60 ms, α = 35°, η = 1, the second with α = 35°, η = 1, T1 = 1180 ms, the third with T1 = 1180 ms, T2 = 60 ms, η = 1 and the fourth with T1 = 1180 ms, T2 = 60 ms, α = 35°. For each set of parameters, the 11 MOLLI points were reordered as a function of TI, and the 3-parameter model was adjusted on the recovery curve. T1 was then calculated (T1 = T1*×(B/A-1)) and compared to the true T1.

The robustness of the standard post-processing approach of the MOLLI sequence was evaluated by simulations, varying either intrinsic tissue parameters (T1/T2) or sequence parameters (α/η). Different datasets were simulated, and the T1 obtained with the 3-parameters fit was compared to the input T1. As depicted by Fig 2, the error made on T1 measurement was quite sensitive to the different parameters. Although the method seemed suitable to measure short T1 values, with high T2, using low nominal flip angle and with good inversion efficiency, discrepancies might become higher than 15% for large-T1 measurement, with high nominal flip angle and poor inversion efficacy (η < 0.9).

### Monte Carlo simulation

**Fig 3:**
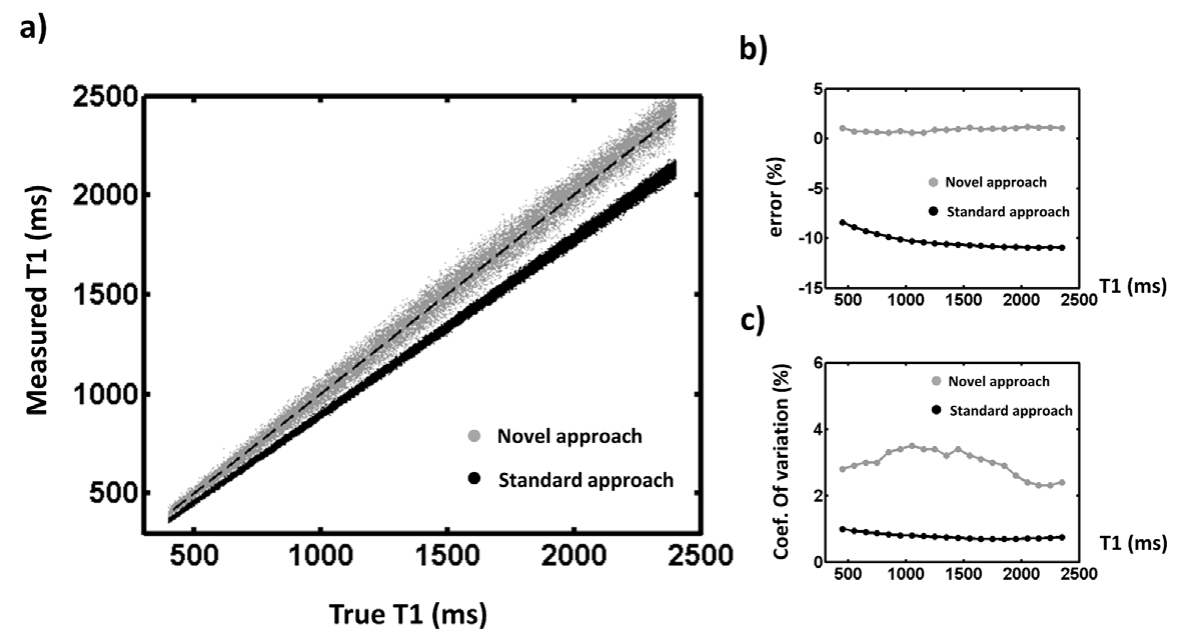
Robustness of both post-processing methods was assessed by Monte Carlo simulations. A large set of data (10^5^) was generated using the Bloch equations simulation and standard *in vivo* parameters: nominal flip angle α_nom_ = 24°, T2_nom_ = 41 ms, 400 < T1 < 2400 ms. For each dataset, the 11 points of the MOLLI sequence were modified with an additional noise corresponding to the experimental SNR. Datasets were then processed either by the standard or the novel post-processing approach using modified T2 and flip angle values (T2_mod_ and α_mod_) randomly generated from standard deviations of T2 and B1+ mapping sequences. T1 values estimated by the two post-processing methods were compared to true T1 values (a). The T1 exploration window was divided into 20 intervals, wherein mean relative errors (b) as well as coefficient of variation of the values (c) were measured.

Fig 3A represents T1 values obtained by Monte Carlo simulations with the two approaches as a function of true T1. As expected, the standard method underestimated longitudinal relaxation times while the novel approach remained consistent for the entire exploration windows (Fig 3B). The standard post-processing approach presented a coefficient of variation (defined by the ratio between the standard deviation and mean value of the measure) of about 1% whereas sensitivity of the proposed method ranged from 2 to 4% (Fig 3C).

### Inversion efficacy

**Fig 4:**
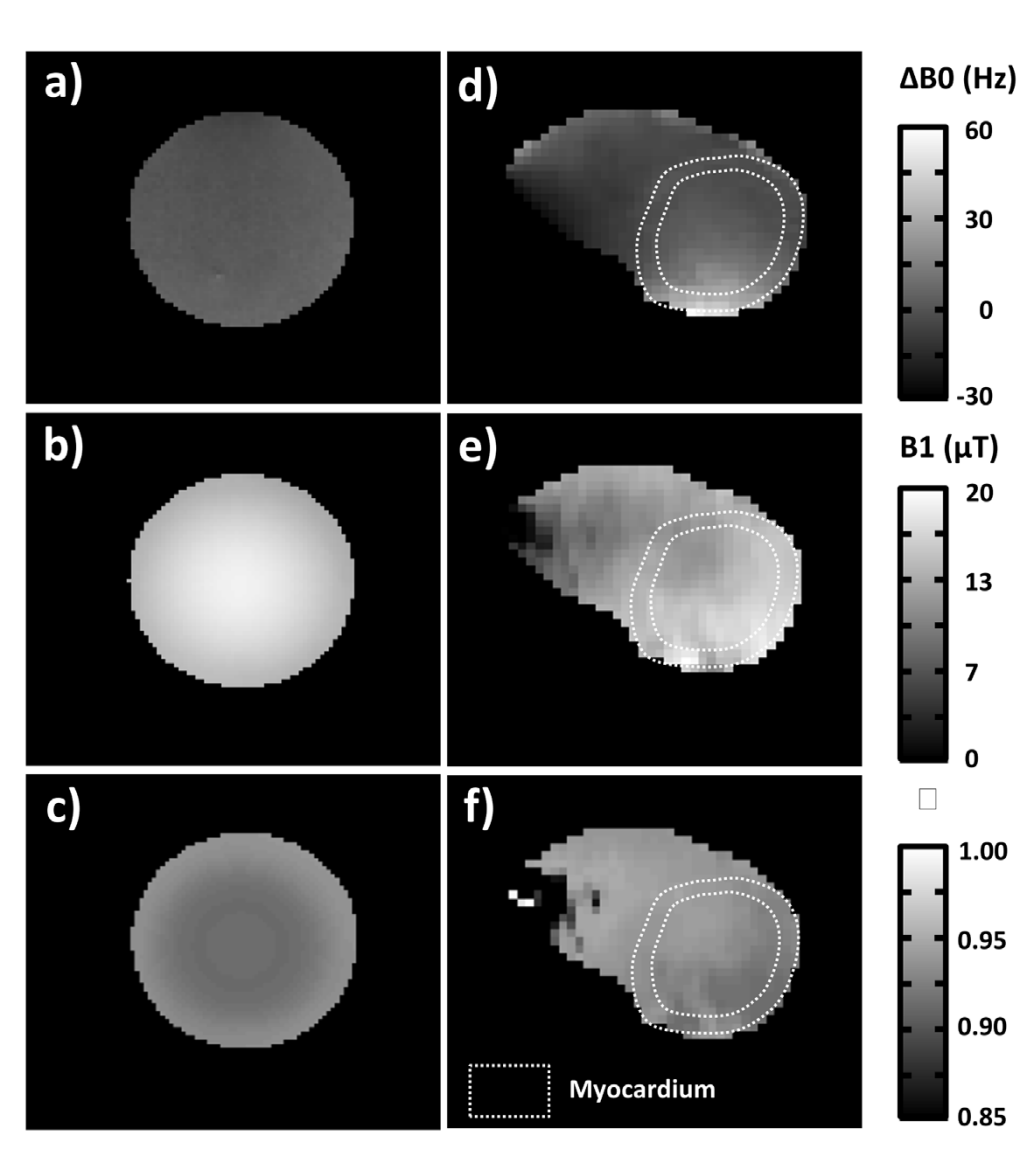
B0 (a,d) and B1+ (b,e) maps acquired respectively on the 2.2L agar phantom and on one of the volunteers. Inversion efficacy maps (c,f) were derived using a Bloch simulation of the 10 ms hyperbolic secant pulse used in the MOLLI sequence as well as B0 and B1+ constraints with a nominal B1 of 16.1μT.

Inversion efficacy η was estimated using a Bloch simulation of the 10 ms hyperbolic secant pulse used in the MOLLI sequence (nominal B1+ = 16.1μT) as well as B0 and B1+ maps acquired *in vitro* and *in vivo* on one volunteer. It can be seen that B0 and B1+ inhomogeneities are significantly higher *in vivo* compared to the phantom (Fig 4A and 4B, 4D and 4E). Nevertheless, even with these constraints, η was found highly homogenous both in the phantom and in the myocardium with a mean value of 0.92±0.01 (Fig 4C–4F). Following post-processing using Bloch-equations simulation was performed with this value of η.

### In vitro validation

**Fig 5:**
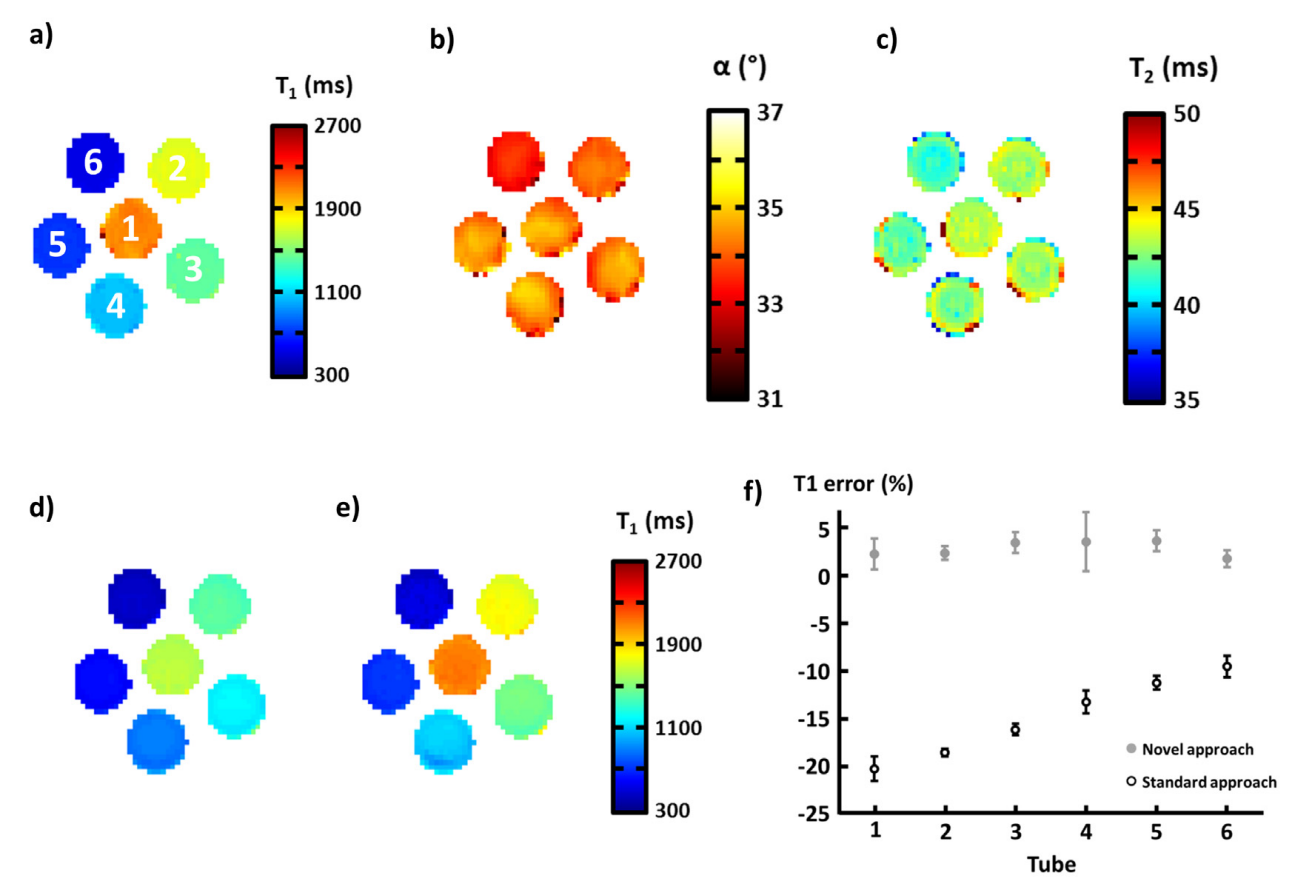
T1 (a), B1+ (b) and T2 (c) maps acquired on Gd-DOTA-doped phantoms using gold-standard sequences. T1 maps derived by the standard post-processing approach of the MOLLI sequence (d) and the novel simulation method (e). Mean relative errors between T1 values derived from gold standard sequence and the two different post-processing approaches of MOLLI sequence (f).

Fig 5A–5C shows T1, T2 and B1+ maps acquired on the phantom with the standard sequences (IR-TSE, Bloch-Siegert GRE, spin echo at different TE). Values (± standard deviations) of the different parameters are summarized in Table 2. The six tubes exhibited homogeneous T2 and B1+ values (approximately 42–43 ms and 34–35° respectively) and a broad distribution of T1 (from 535 ± 6 ms to 2085 ± 32 ms). The MOLLI sequence was acquired during the same imaging session and the novel post-processing approach based on sequence simulation was compared to the standard 3-parameter fitting method for T1 mapping (Fig 5D and 5E).

**Table 2:** Values (± standard deviations) of true T1, true T2 and excitation flip angle parameters measured on the Gd-DOTA doped phantoms by the gold-standard sequence, and T1 values derived from both post-processing methods of the MOLLI sequence.

Tube	1	2	3	4	5	6
**T1 (ms) IR-TSE**	2085 ± 32	1747 ± 6	1422 ± 9	1038 ± 14	706 ± 6	535 ± 6
**T2 (ms) SE**	43.5 ± 0.4	42.9 ± 0.3	42.9 ± 0.4	42.8 ± 0.7	42.1 ± 0.6	41.8 ± 0.6
**α (°) BS**	35.3 ± 0.5	34.7 ± 0.5	35.3 ± 0.5	35.3 ± 0.8	34.8 ± 0.8	33.6 ± 0.6
**T1 (ms) MOLLI standard**	1665 ± 10	1424 ± 4	1193 ± 3	900 ± 2	627 ± 3	484 ± 3
**T1 (ms) MOLLI fit-sim**	2133 ± 10	1789 ± 10	1471 ± 13	1076 ± 34	732 ± 7	544 ± 4

For high T1 values, as already observed in [8] and [14], T1 generated by the standard technique is more than 20% underestimated. On the contrary, the new post-processing approach was quite consistent with the results obtained by the IR-TSE sequence (Fig 5F). Error made with the standard approach was found between 9.5% and 20.1% (depending of the T1), while the maximum deviation was less than 4% using the novel method. Standard deviations were higher on T1 maps derived by the novel method (1.1% against 0.4% for the standard post processing): this was due to a much more complex post-processing pipeline as well as the accuracy of T2 and B1+ parameters acquired by SE and Bloch-Siegert sequences.

**Fig 6:**
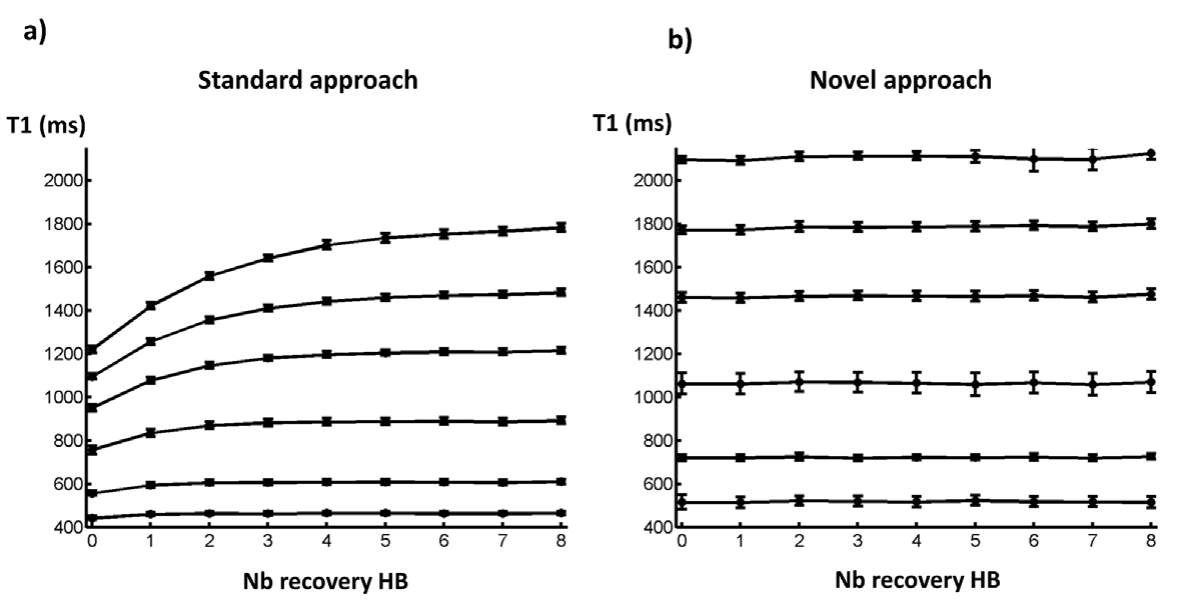
Influence of the number of R-R recovery periods between inversion pulses on T1 values estimated by the standard (a) and the novel (b) post-processing approaches of MOLLI sequence.

As depicted by Fig 6, and contrary to the standard fitting method, T1 values derived by the novel post-processing approach did not vary with the number of recovery periods before each inversion. Differences between T1 measured with an 8 R-R recovery period and no recovery period were less than 2.5% with the proposed method, whereas they were ranging from 4.2 to 28.8% with the standard approach.

### T2 mapping with T2-prepared b-SSFP

T2 values determined by the T2-prepared b-SSFP sequence were compared to the T2 derived from standard SE sequences at different TE on phantoms exhibiting a large range of transverse relaxation times. T2 values determined by the T2-prepared b-SSFP were highly correlated to the gold standard T2 values (R^2^ = 0.998) but were constantly 7% overestimated. For typical myocardium T2 values that are around 45 ms [23], it corresponds to an overestimation of 3.1ms. According to Bloch-equations simulations, it would lead to a T1 underestimation of less than 0.25% for a typical value of 1300ms when the novel post-processing method will be used.

### In vivo experiments

**Fig 7:**
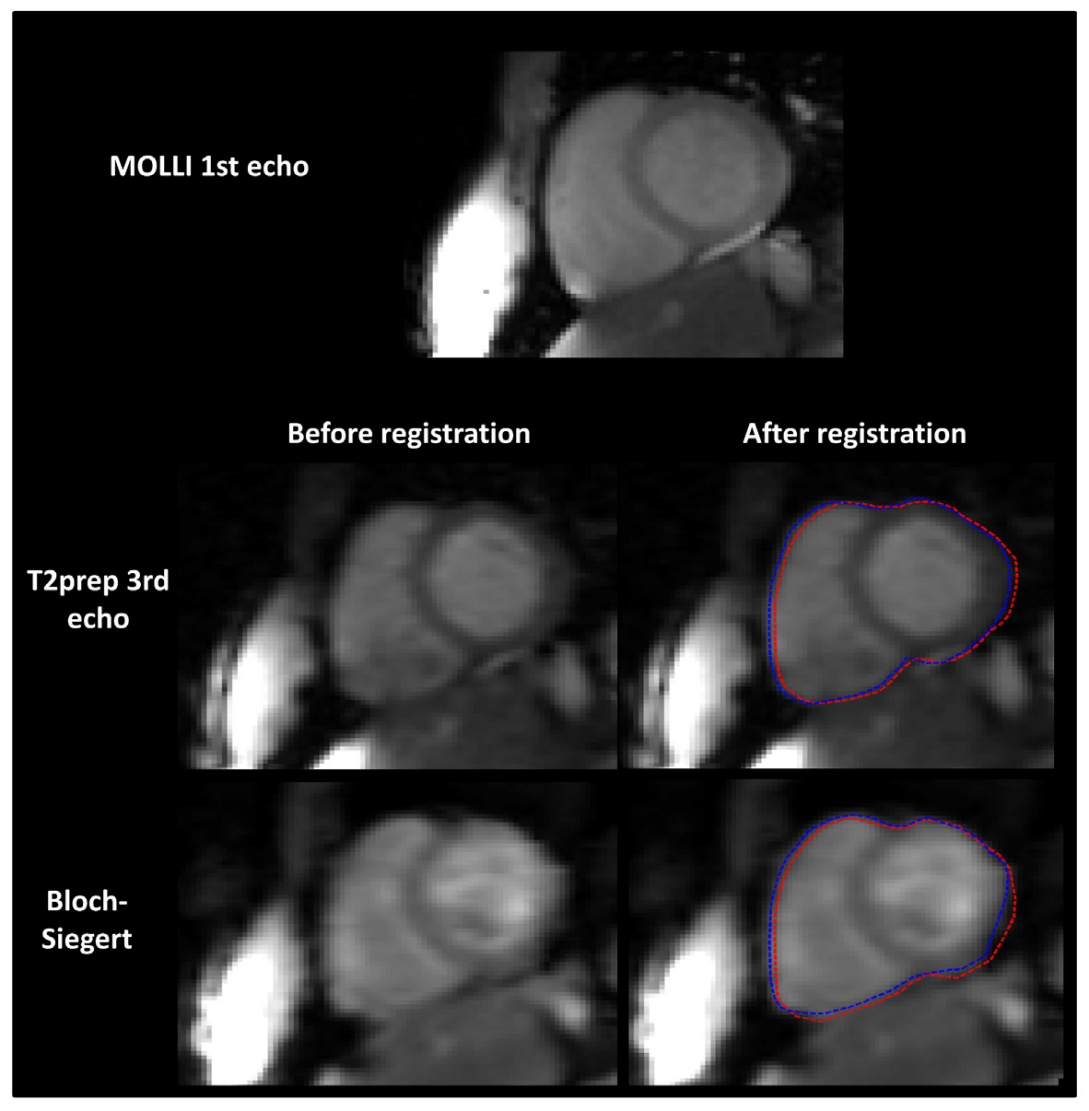
Typical images acquired in vivo on one healthy volunteer with the MOLLI, T2-prepared b-SSFP and Bloch-Siegert sequences. Contours of the heart were manually drawn before and after rigid registration (red and blue dashed lines respectively) and overlaid on the registered images.

Fig 7 shows typical images acquired in vivo with the MOLLI, T2-prepared b-SSFP and Bloch-Siegert sequences. Contours of the heart were manually drawn before and after rigid registration (red and blue dashed lines respectively) and overlaid on the registered images. As depicted by the figure, displacement was minimal and in the range of a few pixels.

**Fig 8:**
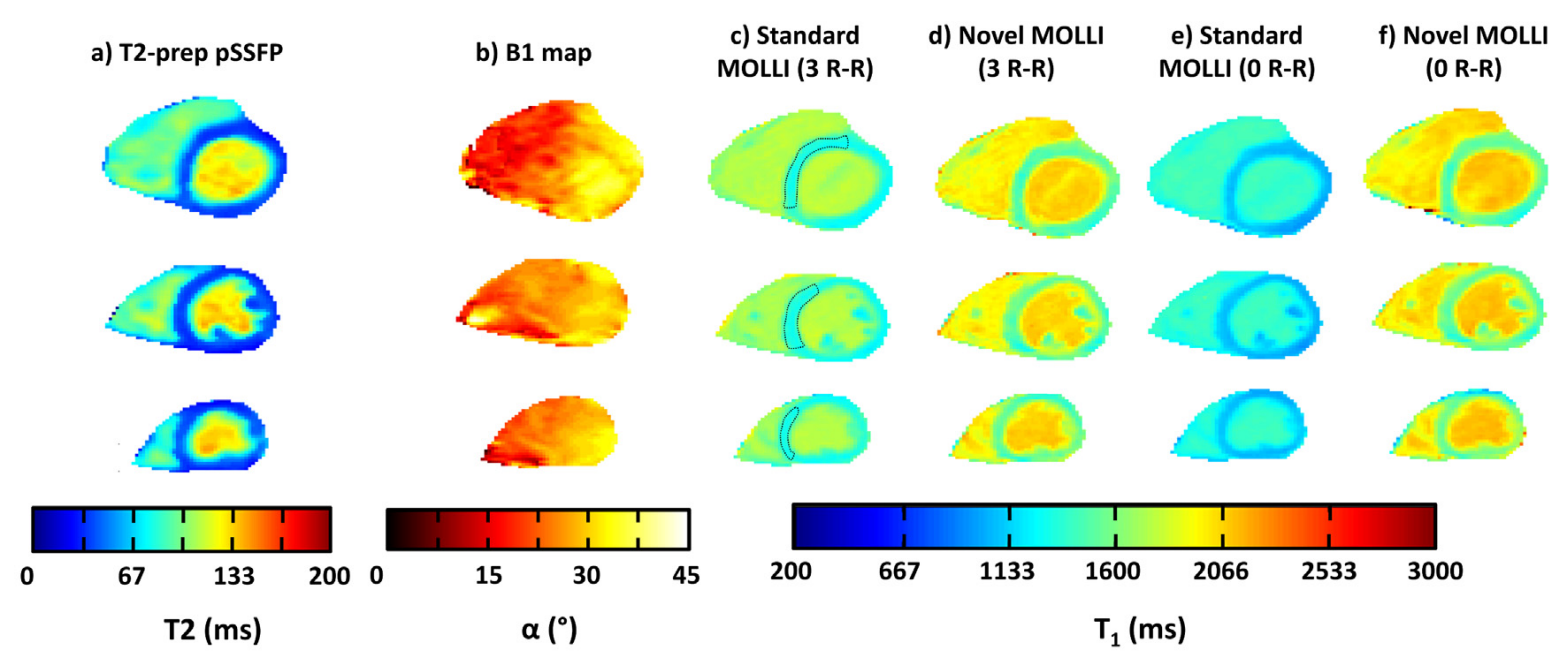
*In vivo* T2 and B1+ maps obtained using T2-prepared TrueFISP and Bloch-Siegert sequences, respectively (a and b). *In vivo* T1 maps generated from standard and novel post-processing approaches of the MOLLI sequence (3HB) (c and d respectively). *In vivo* T1 maps generated from standard and novel post processing of the MOLLI sequence acquired without recovery period before second and third inversions (0HB) (e-f).

**Fig 9:**
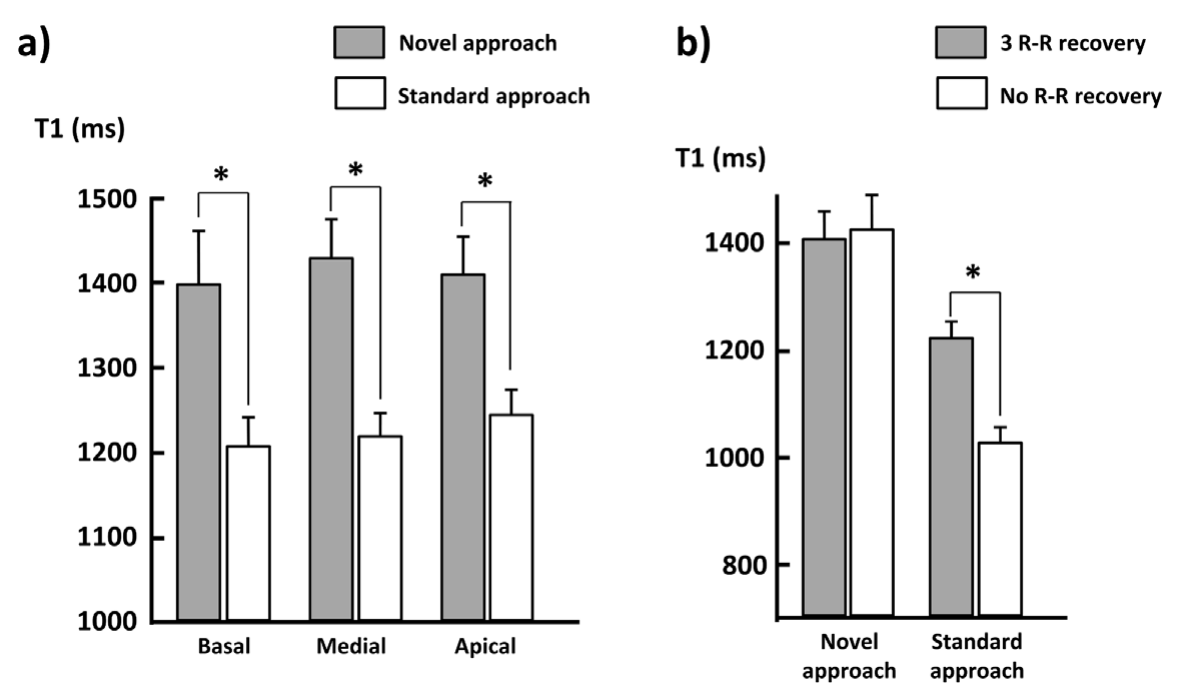
T1 values measured at three levels (basal, medial and apical) of five healthy volunteers myocardium using the standard and the novel post-processing approaches of MOLLI (a). T1 values measured with the two sequence settings for each post-processing method (b). (*: P < 0.01).

In vivo T2 maps (Fig 8A) were homogenous through the myocardium of the five healthy volunteers with a mean value of 40.7±3.0 ms. The segmented/interleaved Bloch-Siegert GRE sequence allowed the acquisition of B1+ maps with limited artifacts induced by cardiac motion (Fig 8B). The mean flip angle value was significantly lower than the prescribed one (α = 35°) for all the subjects. A reproducible pattern of B1+ heterogeneities was observed for the majority of the volunteers: B1+ increases smoothly from the right ventricle to the left. MOLLI sequences were post-processed by both the standard and novel procedure, and therefore two T1 maps were generated at the three slice levels (basal, medial, apical) for each volunteer (Fig 8C and 8D). Mean parameters values (± standard deviation) are summarized in Table 3 for each individual subject. Myocardium T1 values determined by the standard post-processing procedure (1221±30 ms) were once again underestimated compared to the values generated using the simulation procedure (1410±51 ms). The group study revealed that the underestimations were statistically significant (P < 0.01) and respectively equal to 15%, 15% and 14% at the basal, medial and apical levels (Fig 9A). Standard deviations of T1 measures were significantly increased by 91%, 64% and 43% at the basal, medial and apical levels, using the novel post-processing method (p<0.01).

**Table 3:** T2, excitation flip angle and T1 values (± standard deviation) derived from both post-processing methods of MOLLI in the myocardium of five healthy volunteers.

Volunteer		1	2	3	4	5
**T2 (ms)**	**Basal**	43 ± 3	41 ± 4	39 ± 2	38 ± 4	40 ±3
	**Medial**	43 ± 3	41 ± 3	38 ± 2	37 ± 1	38 ± 3
	**Apical**	46 ± 5	42 ± 2	41 ± 4	42 ± 3	42 ± 3
α **(ms)**	**Basal**	22 ± 2	20 ± 5	28 ± 3	23 ± 3	27 ± 3
	**Medial**	19 ± 3	22 ± 2	26 ± 1	26 ± 2	24 ± 3
	**Apical**	19 ± 2	22 ± 2	26 ± 2	24 ± 2	26 ± 2
**T1 standard (ms)**	**Basal**	1239 ± 48	1237 ± 36	1171 ± 26	1193 ± 25	1185 ± 31
	**Medial**	1319 ± 34	1156 ± 26	1230 ± 25	1203 ± 33	1176 ± 22
	**Apical**	1336 ± 42	1208 ± 19	1221 ± 39	1191 ± 26	1255 ± 24
**T1 fit-sim (ms)**	**Basal**	1421 ± 62	1424 ± 72	1374 ± 86	1353 ± 44	1410 ± 52
	**Medial**	1401 ± 35	1484 ± 42	1417 ± 36	1428 ± 52	1398 ± 65
	**Apical**	1395 ± 48	1453 ± 58	1375 ± 36	1413 ± 33	1405 ± 39

MOLLI sequence was also acquired in vivo without recovery period before second and third inversions (Fig 8E and 8F). This particular setting allows shortening the acquisition time to 11 heartbeats. When data are post processed with the standard method, T1 values are dramatically reduced (1025±31 ms) compared to the 17 heart-beats sequence (Fig 9B). On the other hand, when the novel post-processing is applied, T1 values measured within the myocardium remain statistically identical (1429±62 ms).

## Discussion

Systematic errors on T1 values reported by users of the MOLLI sequence are largely due to an imperfect post-processing procedure that does not take into account b-SSFP readouts interleaved with relaxation periods. In this study, we demonstrated both on calibrated phantoms and *in vivo* that a proper simulation of the sequence allowed for an accurate quantification of T1 without interfering with acquisition parameters (e.g. by decreasing the nominal flip angle or the number of phase-encoding steps).

The accurate knowledge of the excitation flip angle performed during b-SSFP readouts represents a critical factor as it provides an a-priori value for the simulation approach. At high field, considering the size of the chest and its tissues composition, B1+ heterogeneities are expected through it and more particularly over the heart. Thus, the applied flip angle can extensively deviate from the nominal value (more than 20% dispersion over the short-axis slices in this study); and consequently the acquisition of a B1+ map becomes mandatory. A wide variety of B1+ mapping methods have already been developed and most of them rely on image amplitude: e.g. signal ratios method [24–26], steady state signals [27], signal nulling at certain flip angles [28], or comparing the amplitude between stimulated and spin echoes [29]. These conventional approaches that rely on resetting the longitudinal magnetization Mz to a known state prior to imaging are quite time consuming and may suffer from cardiac or respiratory motion. In this work, a B1+ mapping sequence based on Bloch-Siegert shift was implemented for *in vivo* imaging. The method is independent of Mz and encodes transmit power in the signal phase of the image. The resulting B1+ maps are insensitive to chemical shift, TR, T1 relaxation, excitation flip angle, and magnetization transfer [21]. Phase based sequences to measure B1 such as Bloch-Siegert might also be prone to motion and flow. This is particularly true when studying a moving organ like the heart. Concerning flow, Dixon et al. [30] have performed Bloch-Siegert experiments on phantom with a laminar flow of 0.5m/s. Even if they observed a change in the absolute value of pixel phase when flow was on, it did not results in changes in the B1 map provided after processing. For motion artifacts, acquisitions were interleaved and performed during breath hold to avoid respiratory movements. Furthemore, readouts were segmented to reduce echo train duration to 200 ms and fit in the same quiescent windows of the cardiac cycle than the MOLLI b-SSFP trains. Lau et al. demonstrated that the Bloch-Siegert sequence can even be improved by using flow-insensitive spiral readout gradients [31].

Inversion efficiency also represents a major confounder when T1 maps are directly derived from MOLLI sequence with the 3-parameters fitting as it was shown that imperfect inversion may lead to large T1 underestimation. In this study, η was estimated using a Bloch simulation of the MOLLI inversion pulse. It revealed that inversion efficiency was close to 0.9. As adiabatic inversion was applied, η was homogenous through space and was not much affected by B1+ heterogeneities. This value of 0.9 was in good agreement with the study of Kellman et al. [18], and justified the use of a single η for the rest of the study.

The need for additional B1+ and T2 sequences is currently the major limitation of our novel method. While T2 maps are sometimes acquired during an MR cardiac exam and provide information about the potential presence of myocardial edema (e.g. during the acute phase of infarction), B1+ maps are rarely performed and will lengthen the protocol duration. In the present study, a single-slice high-resolution B1+ map was obtained during a 16-heart-beat breath-hold. The total duration of the procedure (shimming and acquisition of basal, medial and apical B1+ maps) took approximately 6 minutes. While applied in clinical protocols, this block could be performed few minutes after Gd-DOTA injection during the resting period of 10–15 minutes before late gadolinium enhancement imaging, since the B1+ mapping sequence is insensitive to T1. Moreover, acquisition time for the B1+ maps at the three levels could be largely reduced to shorter breath-holds by using parallel imaging, degrading spatial resolution or using a more efficient readout approach (EPI, TurboFLASH,…).

The accuracy of the measurement of T2 and excitation flip angle α will strongly determine the sensitivity of the post-processing method based on Bloch equations simulation. In particular, it was shown that standard deviations of T1 values are significantly higher using the simulation method compared to the standard 3-parameter fitting approach. Results given by the latter will mainly depend on the signal-to-noise ratio (SNR) of the 11 data points of the MOLLI sequence, whereas the simulation approach sensitivity will be driven by SNR as well as B1+ and T2 confidence intervals. Monte Carlo simulations were performed using confidence intervals for α and T2 given by standard deviations observed *in vivo* for both parameters. They showed that coefficient of variation are significantly increased when MOLLI data are processed by the simulation approach. The sensitivity of the proposed reconstruction method would certainly benefit from an additional optimization of T2- and B1+-mapping sequences. Moreover, as the proposed method requires the acquisition of additional NMR sequences than MOLLI, it might be affected by the specific artifacts related to these scans or by poor registration if displacement between images become too important. As Bloch Siegert sequence is a gradient echo acquisition, it may be prone to susceptibility artifacts. Over the 5 healthy volunteers scanned in the study, susceptibility artifact were commonly detected at the border of the right ventricle, leading to biased flip angle measurement at these locations. Nevertheless, on the left ventricle, which is the most frequently assessed region of interest in cardiac studies, no susceptibility artifacts were observed for all of the volunteers.

Various methods were recently presented for cardiac T1 mapping. A shortened version of MOLLI with conditional curve fitting (ShMOLLI) was proposed as a means of shortening the breath-hold [32]. ShMOLLI sequence has demonstrated more insensibility to heart rate variability but at the cost of a loss of precision due to a lower number of fitted data points. Furthermore, as MOLLI sequence, ShMOLLI is also biased by the same confounders (T2 decay during b-SSFP train, imperfect inversion) to estimate long T1 values. With minor modifications, the proposed method can be applied to post-process ShMOLLI data, and may be adaptable to any version of MOLLI-derived sequence. For example, in the present study, we demonstrated that MOLLI could be acquired without recovery period before the second and third inversions without corrupting reconstructed T1 maps. This allowed reducing acquisition time of the sequence while keeping 11 points along the T1 recovery curve. Saturation recovery methods (as SASHA) have also been developed for cardiac T1 mapping [33]. A 3-parameter model for data fitting was proposed to make the T1 measurement insensitive to T2 and to saturation efficacy. Nevertheless, this method is based on the acquisition of images shortly after magnetization saturation and is particularly prone to artifacts since these images generally have low SNR. SASHA has the potential to provide more accurate T1 measurement that is less sensitive to MT as well as other factors [34] but is noisier, and have not demonstrated yet the same level of reproducibility than MOLLI-derived sequences [35].

When compared to existing literature at 3T, *in vivo* T1 values measured with the standard post-processing of the MOLLI sequence (1221±31ms) are in good agreement with those measured with the same type of sequence and post-processing (MOLLI or shMOLLI). Piechnik et al. [32] found a T1 of 1169±45 using the shMOLLI approach, von Knobelsdorff-Brenkenhoff et al. [23] measured a mean T1 of 1165ms and Lee et al. [14] a T1 of 1315±39ms with the MOLLI sequence. The mean T1 derived by the novel approach (1410±51ms) is significantly higher. Nevertheless, it is consistent with the few studies performed with other types of acquisitions. Stanisz et al. [36] measured a myocardium T1 of 1471±31ms using a more standard inversion recovery sequence and Clique et al. [37] a T1 = 1341±42ms with a variable flip angle 3D-FSPGR sequence. Concerning in vivo T2 values, the mean value measured in our study (40.7±3.0ms) is consistent with the T2 = 38.5±4.5ms observed by Van Heeswijk et al. [38] and T2 = 45.4±1.4ms measured by von Knobelsdorff-Brenkenhoff et al. [23].

Improved accuracy in the quantification of high T1 values might potentially improve diagnostic value in certain disease, when assessed without contrast agent injection. In Anderson-Fabry disease for example, the slight T1 decrease induced by intracellular lipid disorganization [13] might be more readily observed and quantified using the novel post-processing method. Furthermore, determination of extracellular volume fraction relies on the measurement of blood and myocardial tissues T1 before and after Gd-DOTA injection [7]. At 3T, native T1 values are around 1900 ms and 1300 ms for the blood and the myocardium, respectively, and are largely underestimated with the standard post-processing approach. Given the improved accuracy for native T1 times of the proposed method, the accuracy of ECV estimation can be increased.

At higher magnetic field, B1+ inhomogeneities will even be more important across the entire myocardium than at 3T. T1 maps derived by the 3-parameter-fitting method could therefore present a pattern corresponding to these inhomogeneities that will certainly prevent the radiologist to establish a robust diagnosis. On the contrary, artifacts will be mitigated on the T1 maps obtained by the simulation method. At the cost of additional acquisitions, this approach might be suitable in clinical research protocols for accurate tissue characterization. In conclusion, this work showed that the standard post-processing of MOLLI acquisitions has several limitations which might cause T1 quantification inaccuracies, especially at high field when B1+ homogeneity through the entire heart will no longer be possible. We proposed a novel approach based on Bloch equations simulation that take into account T2, B1+ and inversion efficacy to derive accurate T1 maps of the left myocardium from MOLLI experimental data. Future work will investigate off-resonance and magnetization transfer effects and the ability to take them into account within the novel post-processing method, as it has recently been shown that they might also cause T1 variations with the MOLLI sequence [39,40].

## Appendix

### Full description of matrix and vectors used in the Bloch simulation

Bloch simulations for the MOLLI sequence were implemented using the framework described in details in [17]. They take into account RF pulses, free precession, T1 and T2 relaxation.

RF nutation about the x-axis of an angle α is represented by a multiplication by the matrix:

$$R_{\alpha }=\left[\begin{array}{ccc} 1 & 0 & 0\\ 0 & \text{cos}\;\alpha & \text{sin}\;\alpha \\ 0 & -\text{sin}\;\alpha & \text{cos}\;\alpha \end{array}\right]$$ (7)

Free precession during a period t corresponds to a multiplication by the rotation matrix:

$$P\left(t\right)=\left[\begin{array}{ccc} \text{cos}\left(2\pi \Delta ft\right) & \text{sin}\left(2\pi \Delta ft\right) & 0\\ -\text{sin}\left(2\pi \Delta ft\right) & \text{cos}\left(2\pi \Delta ft\right) & 0\\ 0 & 0 & 1 \end{array}\right]$$ (8)

where Δf is the off-resonance factor.

T1 and T2 relaxation over a time t can be represented by a multiplication by the matrix:

C(t)=[e−tT2000e−tT2000e−tT1] (9)

and the addition of the vector:

$$D\left(t\right)=\left(I-C\left(t\right)\right)\left[\begin{array}{c} 0\\ 0\\ m_{0} \end{array}\right]$$

where **I** is the identity matrix.[35]
